# Modulation of Conjunctival Goblet Cell Function by Inflammatory Cytokines

**DOI:** 10.1155/2013/636812

**Published:** 2013-12-17

**Authors:** L. Contreras-Ruiz, A. Ghosh-Mitra, M. A. Shatos, D. A. Dartt, S. Masli

**Affiliations:** ^1^Department of Ophthalmology, Boston University School of Medicine, Boston, MA 02118, USA; ^2^Department of Ophthalmology, Harvard Medical School, Schepens Eye Research Institute and Massachusetts Eye and Ear, Boston, MA 02114, USA

## Abstract

Ocular surface inflammation associated with Sjögren's syndrome is characterized by a loss of secretory function and alteration in numbers of mucin secreting goblet cells. Such changes are a prominent feature of ocular surface inflammatory diseases and are attributed to inflammation; however, the exact effect of the inflammatory cytokines on conjunctival goblet cell function remains largely unknown. In this study, we developed a primary culture of mouse goblet cells from conjunctival tissue and evaluated the effects on their function by inflammatory cytokines detected in the conjunctiva of mouse model of Sjögren's syndrome (Thrombospondin-1 deficient mice). We found that apoptosis of goblet cells was primarily induced by TNF-**α** and IFN-**γ**. These two cytokines also inhibited mucin secretion by goblet cells in response to cholinergic stimulation, whereas IL-6 enhanced such secretion. No changes in secretory response were detected in the presence of IL-13 or IL-17. Goblet cells proliferated to varying degrees in response to all the tested cytokines with the greatest response to IL-13 followed by IL-6. Our results therefore reveal that inflammatory cytokines expressed in the conjunctiva during an ocular surface disease directly disrupt conjunctival goblet cell functions, compromising the protective function of tears, thereby contributing to ocular surface damage.

## 1. Introduction

Mucin-secreting goblet cells are widely distributed throughout mammalian mucosal surfaces, such as the gastrointestinal, urogenital, and respiratory tracts, where they play a key role in hydrating, lubricating, and clearing pathogens from the underlying epithelium [1]. The importance of goblet cells as major producers of mucins is well established, with critical emphasis placed on the number of functional goblet cells and on the amount and rate at which they synthesize mucins. In fact, alterations in goblet cell numbers and mucin secretion are prominent features of mucosa associated diseases, with increased goblet cell numbers and hypersecretion in conditions such as asthma or cystic fibrosis [2, 3], and mucin depletion and diminished goblet cell density in intestinal diseases such as inflammatory bowel disease or ulcerative colitis [4, 5].

In the eye, goblet cells are the principal secretory cell in the conjunctival epithelium, where they function in lubricating the ocular surface epithelia during the blink response stabilizing the tear film, and as a physical barrier to pathogen penetration [6]. Alterations in goblet cell secretion lead to an unstable tear film and a vulnerable ocular surface. Goblet cell loss has been reported in several inflammatory diseases of the ocular surface, including Stevens-Johnson syndrome, ocular mucous membrane pemphigoid, alkali burn, neutrophilic keratitis, graft-versus-host-disease, and Sjögrens's syndrome (SS) [7–9].

Although the mechanisms leading to goblet cell changes in the eye are not entirely understood, evidence in other mucosal tissues suggests that inflammation may have an important contribution. It is known that IL-13 is involved in lung goblet cell hyperplasia and mucus hypersecretion [10], while IFN-*γ* inhibited IL-13-induced goblet cell hyperplasia in a mouse model of airway inflammation [11] and it is a potent inhibitor of mucin secretion in a human colonic goblet cell line [12]. Although both IL-13 and IFN-*γ* have been reported in ocular inflammatory conditions it is not known whether conjunctival goblet cells respond similarly to those in the lungs with mucus hypersecretion or with inhibition of secretion [13, 14]. It was also previously reported that overexpression of IL-17A induces respiratory mucous metaplasia [15]. However, the role of inflammation in conjunctival goblet cell function has remained unaddressed, partly due to lack of *in vitro* cell cultures that allow study of goblet cells without altering their phenotype and function. Therefore, we have developed a primary culture of mouse goblet cells from conjunctival tissue to evaluate the effects of inflammatory cytokines on goblet cells with respect to processes such as mucin secretion, proliferation, and apoptosis.

We have previously described extensively an autoiummune SS-associated ocular phenotype in Thrombospondin-1 (TSP-1) deficient mice that resembles the changes detectable in SS patients [16]. These mice spontaneously and progressively develop inflammation in the conjunctiva, with appearance of inflammatory infiltrates, tissue expression of Th1 and Th17 inflammatory cytokines, along with the development of inflammatory T cell effectors in their draining lymph nodes [17]. Similar to SS patients, significant changes in goblet cell numbers are detected in TSP-1 deficient mice along with reduced tear mucin level.

Our primary purpose in this study was to evaluate whether inflammation in TSP-1 deficient conjunctiva disrupts the functions of goblet cells. We used cultured goblet cells from mouse conjunctiva to study the effect of inflammatory cytokines detected in TSP-1 null conjunctiva on secretory and proliferative properties of goblet cells. The studies described herein indicate that mouse goblet cells, as shown previously with rat and human goblet cells [18, 19], can be isolated from mouse conjunctiva retaining *in vivo* characteristics of mouse goblet cells, and that the proinflammatory cytokines expressed in TSP-1 null conjunctiva induce their proliferation in varying degrees. Greatest proliferation was induced by IL-13 with IL-6 following closely. Both TNF-*α* and IFN-*γ* induced goblet cell apoptosis while inhibiting mucin secretion induced by cholinergic stimulation. Contrary to this effect IL-6 enhanced such mucin secretion by goblet cells. Our results therefore reveal that inflammation can directly disrupt conjunctival goblet cell functions resulting in an altered tear composition with a compromised protective function, which contributes to ocular surface damage.

## 2. Materials and Methods

### 2.1. Mice

C57BL/6 (H-2b) mice, 4 to 22 weeks old, were purchased from Charles River Laboratories (Wilmington, MA). TSP-1 null mice (C57BL/6 background), originally received from Dr. J. Lawler (BIDMC, Harvard Medical School, Boston, MA) were bred in-house in a pathogen-free facility at Schepens Eye Research Institute, Boston, MA. All experiments were conducted in accordance with institutional guidelines and ARVO Statement for the Use of Animals in Ophthalmic and Vision Research.

### 2.2. RT-PCR

Total RNA was isolated from conjunctivas harvested from WT or TSP-1 null mice (6, 8, and 12 weeks, *n* = 3 to 5) using TRIzol Reagent (Life Technologies, Carlsbad, CA) according to the manufacturer's instructions. cDNA was synthesized by reverse transcribing RNA using oligo (dT) and M-MLV RT (Promega, Madison, WI). Real-time PCR assay was performed on the Eppendorf Realplex2 system (Eppendorf AG, Hamburg, Germany) using SYBR Green PCR Master Mix (Applied Biosystems, Carlsbad, CA) to determine relative quantitative expression levels of Interleukin (IL)-13 and GATA3 genes. IL-13 primers (F-5′-AGAATGGCCTGTTACACTCA-3′ and R-5′-TTTCCGGTTTCTAGTTTGA-3′), GATA3 primers (F-5′-GCCTGGCGCCGTCTTGATA-3′ and R-5′-CCCGGTCAGATTGCG TAGCTC-3′), and glyceraldehyde-3-phosphate dehydrogenase primers (F-5′-CGAGAATGGGAAGCTTGTCA-3′ and R-5′-AGACACCAGTAGACTCCACGACAT-3′) were used. Amplification reactions were set up in quadruplicates with the thermal profile: 95°C for three minutes, 40 cycles at 95°C for ten seconds, 53°C for ten seconds, and 72°C for ten seconds. To verify the specificity of the amplification reaction, a melting curve analysis was performed. Fluorescence signal generated at each cycle was analyzed using system software. The threshold cycle values were used to determine relative quantification of gene expression with glyceraldehyde-3-phosphate dehydrogenase as a reference gene.

### 2.3. Isolation and Culture of Goblet Cells

Goblet cells from mouse conjunctival pieces were grown in organ culture, as described previously for rat and humans [18, 19]. Conjunctival tissues were excised from 4- to 22-week-old male mice and placed into Hank's balanced salt solution (Lonza, Walkersville, MD). Tissues were finely minced into small pieces that were anchored onto scored 24-well culture plates. Approximately 65 to 90 explants were obtained from each animal and four pieces of tissue were anchored per culture well. The culture dishes contained just enough medium to cover the bottom of the well so that the tissue would receive nutrients through surface tension. Explants were fed every other day with RPMI-1640 medium (Lonza, Walkersville, MD) supplemented with 10% heat-inactivated fetal calf serum (Thermo Fisher Scientific, Waltham, MA), 1 mM sodium pyruvate, 10 mM HEPES, 100 *μ*g/mL penicillin-streptomycin, and 1X nonessential amino-acid mixture (Lonza, Walkersville, MD) and grown under routine culture conditions of 5% CO_2_ at 37°C. Cells were permitted to grow from the tissue explant for 14 days until reaching 85% confluence; and then the explant was removed and discarded.

### 2.4. Immunofluorescence

Cultured conjunctival goblet cells were fixed in ice-cold methanol and examined for the presence of cytokeratin (CK)-4, CK-7, and MUC5AC. Cells were incubated at room temperature for one hour with blocking buffer composed of phosphate-buffered saline (PBS) with 2% bovine serum albumin and 0.02% Triton-X (all from Sigma-Aldrich). Afterwards, the cells were incubated overnight at 4°C with the primary antibodies anti-CK-7 (10 *μ*g/mL), which recognizes a goblet cell-specific keratin [20], anti-CK-4 (10 *μ*g/mL), a specific marker for stratified, squamous, nongoblet epithelial cells [20], and MUC5AC (2 *μ*g/mL), specific for mucin produced by goblet cells [21] (all from Abcam, Cambridge, MA). After rinsing in PBS, the cells were incubated with AlexaFluor 488- or 568-conjugated secondary antibodies (6 *μ*g/mL; Life Technologies, Carlsbad, CA) for one hour at room temperature, washed, and mounted for microscopy examination in a Nikon Eclipse E-800 fluorescence microscope (Nikon, Melville, NY).

### 2.5. Flow Cytometry

Cultured conjunctival goblet cells were stained with eFluor 780-conjugated Fixable Viability Dye (eBioscience, San Diego, CA). Intracellular MUC5AC was evaluated with anti-MUC5AC antibody (Abcam) and DyLight 649-conjugated secondary antibody (Abcam) using an intracellular staining kit (eBioscience) as per the manufacturer's instructions. Fluorescence-labeled cells were analyzed using BD LSRII Flow Cytometer (BD Bioscience, San Jose, CA). Further analysis of the data was performed using FlowJo v9.4.10 software (Tree Star, Inc., Ashland, OR).

### 2.6. Cytokine Treatments

To evaluate the effect of inflammatory conditions on goblet cell function, cultured conjunctival goblet cells were grown for 14 days until 85% confluence was reached. Prior to any cytokine exposure, cells were maintained for 24 h in serum-free medium. After this, cultured conjunctival goblet cells were treated with 10 ng/mL of the recombinant cytokines IL-13, IFN-*γ*, TNF-*α*, IL-6, and IL-17A (R&D Systems, Minneapolis, MN) for 24 h. Viability of cells in response to stimulatory cytokines (63% IL-13-treated, 66% in IL-6-treated) was comparable to that in response to inhibitory cytokines (65% IFN-*γ*-treated, 71% TNF-*α*-treated) and untreated control cells (66%).

### 2.7. MUC5AC Secretion: ELISA

Treated and control cultures of conjunctival goblet cells were stimulated with the cholinergic agonist carbachol (10^−3 ^M, Sigma-Aldrich) for one hour and then MUC5AC secretion was measured in the supernatants using the Mucin-5 Subtype AC (MUC5AC) ELISA kit (TSZ ELISA, Waltham, MA) according to the manufacturer's instructions. The cells were also collected and cell homogenates were analyzed for the total amount of protein using the BCA Protein Assay Kit (Pierce, Rockford, IL). MUC5AC secretion was normalized to total protein in the homogenate, and the result was presented as MUC5AC ng/mg of cellular protein.

### 2.8. Cell Proliferation and Apoptosis

Treated and control cultured goblet cells were pulsed with the thymidine analog, 5-bromo-2-deoxyuridine (BrdU, 1 mM) (EMD Millipore, Billerica, MA) for 24 h. Afterwards, the cells were fixed and permeabilized using an intracellular staining kit (eBioscience), treated with DNase (Sigma-Aldrich) to expose incorporated BrdU and stained with a biotin-conjugated anti-BrdU antibody (1 *μ*g/10^6^ cells; Invitrogen) for one hour at 4°C. After rinsing in PBS, cells were incubated with streptavidin-FITC-conjugated reagent (1 *μ*g/10^6^ cells; BD Bioscience) for one hour at 4°C and stained with the DNA dye 7-amino-actinomycin-D (7-AAD) (eBioscience). BrdU content (FITC) and total DNA content (7-AAD) were determined using BD LSRII Flow Cytometer and FlowJo software, and the percentage of cells in each of the G_0_-G_1_, S, G_2_-M phases and apoptotic cells were calculated.

### 2.9. Statistical Analysis

Student's *t*-test was used to determine significant differences between mean values of experimental and control groups. Error bars in figures represent ± standard error of the mean (SEM). *P* < 0.05 was considered statistically significant.

## 3. Results

### 3.1. Th2 Inflammatory Response in TSP-1 Null Conjunctiva

**Figure 1:**
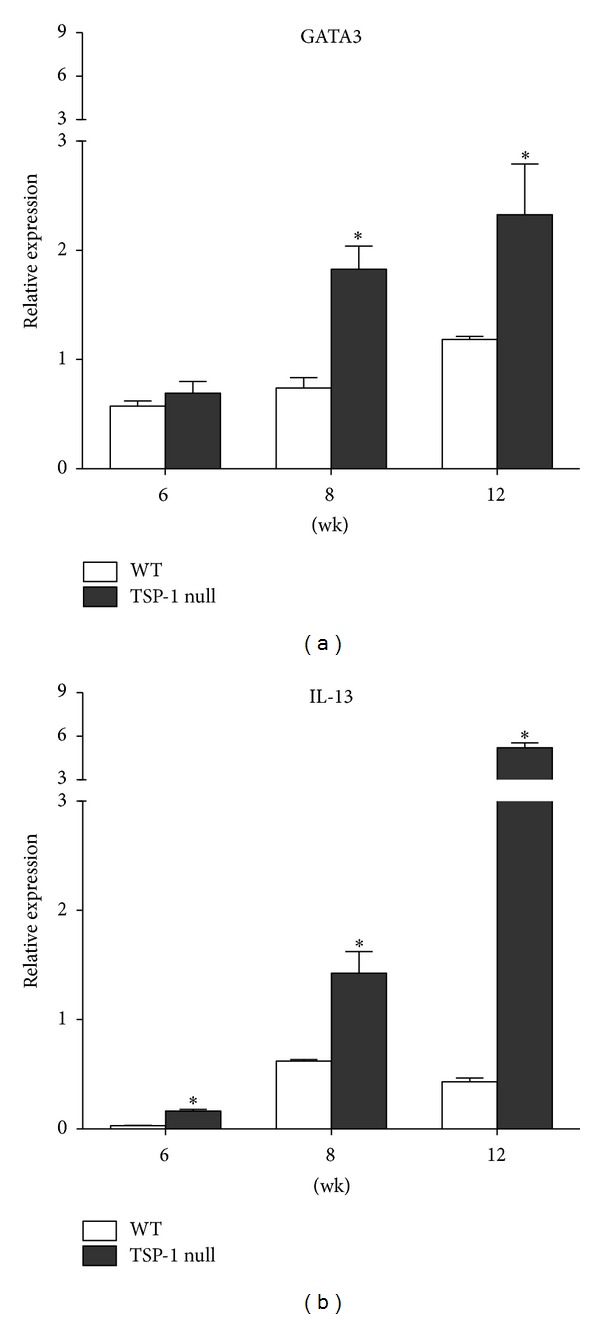
Th2-type inflammatory response is increased in TSP-1 null conjunctiva. Conjunctiva tissues were collected from WT and TSP-1 null mice at 6, 8, and 12 weeks. Extracted RNA was analyzed in a real time PCR assay to determine the levels of message for the Th2 cytokine IL-13 and the Th2-associated transcription factor GATA3 *P < 0.05.

In human SS pathology a dynamic balance between Th1 and Th2 cytokines was reported with the latter found to prevail in low-grade infiltration of salivary glands [22]. Similarly Th2-mediated pathology was reported in the MRL/lpr mouse model of SS [23]. To determine if the spontaneous ocular surface inflammation noted in TSP-1 deficient mice involves Th2 cytokines, we examined conjunctival expression of the transcription factor GATA-3, an essential mediator of these cytokines. We also correlated this expression with that of a representative Th2 cytokine IL-13. Real-time PCR analysis on RNA isolated from conjunctiva derived from WT or TSP-1 null mice at ages 6, 8, and 12 weeks was performed to study the expression levels of GATA-3 and IL-13. Although no differences in GATA3 expression were detectable at 6 weeks of age, at 8 and 12 weeks the expression was significantly increased in TSP-1 deficient conjunctiva compared with the WT control tissues (Figure 1(a)). Consistent with this result, overexpression of IL-13 was detected in TSP-1 null conjunctiva at all ages compared to the aged-matched WT control tissues (Figure 1(b)). Together these results suggest involvement of Th2-mediated pathology in the ocular surface inflammation in TSP-1 null mice similar to that reported by others in SS.

### 3.2. Primary Mouse Conjunctival Goblet Cell Culture

It has been reported that IL-13 is involved in lung goblet cell hyperplasia and mucus hypersecretion [10]. Increased expression of IL-13 in TSP-1 null conjunctiva is consistent with increased goblet cell numbers detected in the earlier stages of ocular surface inflammation between 8 and 12 weeks of age [16]. However, by 15 weeks of age a significant decline in filled goblet cells is detected in TSP-1 null conjunctiva as compared to age-matched WT controls [17]. To allow for an investigation of a direct effect of conjunctival cytokines, if any, on goblet cell proliferation or mucin secretion, we established *in vitro* cultures of goblet cells derived from conjunctival tissue explants based on a method originally described using rat and human tissue [18, 19].

**Figure 2:**
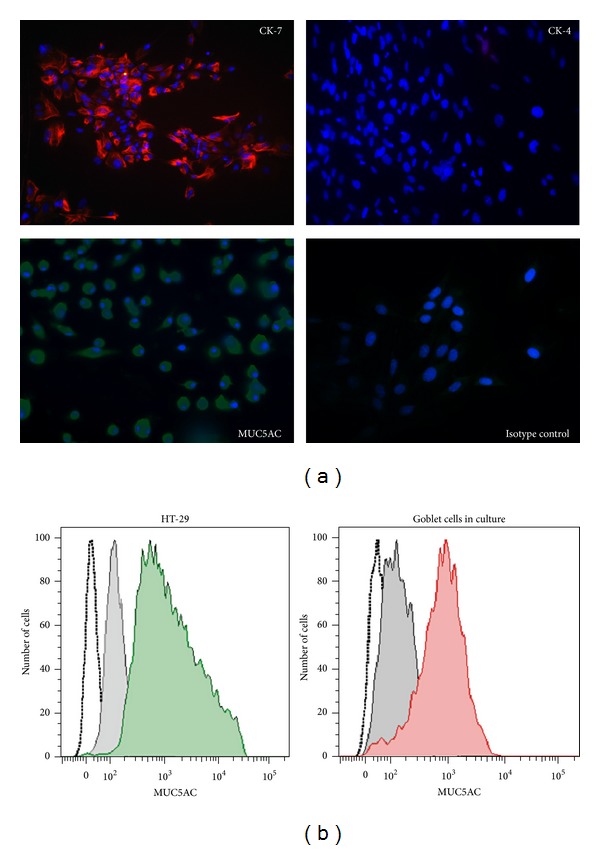
Primary mouse goblet cell cultures express goblet cell specific markers. (a) Cells were grown from WT conjunctival explants for 14 days, and the expression of goblet cell specific (CK-7-red and MUC5AC-green) and stratified squamous cell specific (CK-4-red) markers was analyzed by immunofluorescence. Nuclei were counterstained with DAPI (blue). Magnification = ×20. (b) Flow cytometric analysis of goblet cells for the expression of MUC5AC. Unstained cells (empty histogram), isotype controls (filled grey histogram), colonic HT-29 cells (filled green histogram), and cultured goblet cells (filled red histogram).

Cells were grown from WT conjunctival explants. After 14 days of culture, epithelial morphology of most of the cells was apparent with the presence of distinct secretory granules. To determine if these were goblet cells extensive characterization with goblet cell-specific markers was performed. These included goblet cell derived soluble mucin, MUC5AC, and an intermediate filament associated solely with goblet cells, cytokeratin 7 (CK-7). Any presence of stratified squamous epithelial cells was ruled out by staining for their marker cytokeratin 4 (CK-4). As seen in Figure 2(a), most cells in culture stained positively for goblet cell specific markers MUC5AC and CK-7 with a detectable intense cytosolic signal for both these markers. On the other hand, no signal was detected when cells were stained for CK-4 resembling the isotype control staining with no fluorescence signal. Furthermore, flow cytometric analysis of cultured cells stained for intracellular MUC5AC was performed to establish a quantitative estimate of goblet cells in the culture. As shown in Figure 2(b), a strong signal was detected in positive control HT-29 cell line as all the cells stained positively for MUC5AC, and similarly most cells (>85%) from primary cultures were positively stained for MUC5AC. These results confirmed that almost all cells in our conjunctival explant-derived primary cultures were goblet cells that can be further used in an *in vitro* assay.

### 3.3. Deficiency of TSP-1 in Goblet Cells Does Not Alter Their Ability to Secrete MUC5AC in Response to Cholinergic Stimulation

**Figure 3:**
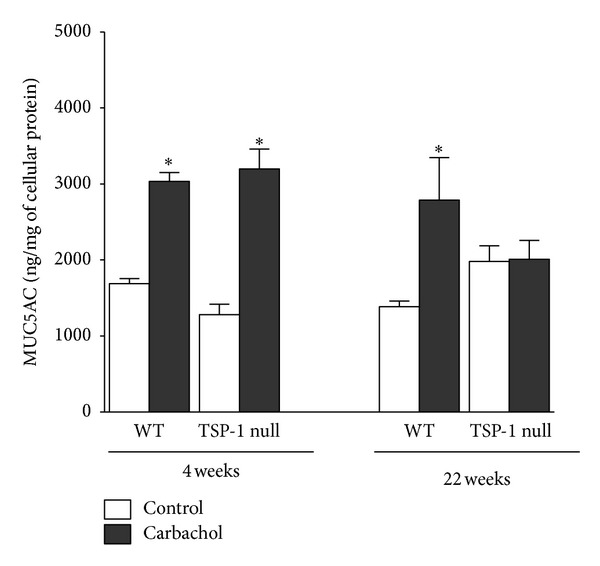
Conjunctival inflammation associated with TSP-1 deficiency prevents carbachol-mediated MUC5AC secretion. Cultured goblet cells were grown from conjunctival explants of WT and TSP-1 null mice at 4 and 22 weeks of age. Goblet cells were stimulated with the cholinergic agonist carbachol, and MUC5AC secretion evaluated by ELISA in the supernatant. *P < 0.05.

Cultured goblet cells respond to *in vitro* cholinergic stimulation induced by carbachol by releasing their vesicular content of MUC5AC into the culture supernatant. In rat and human goblet cell cultures this was determined using an assay that detects glycosylated carbohydrates [18, 19]. We used MUC5AC ELISA to assess the secretory function of WT and TSP-1 null goblet cells in primary cultures. These cells were generated from either 4-week or 22-week-old mice. Upon carbachol stimulation MUC5AC in the culture supernatant was compared to that collected from unstimulated cells. As shown in Figure 3, both 4-week-old WT and TSP-1 null conjunctiva-derived primary cultures of goblet cells responded to carbachol stimulus by secreting significantly increased amounts of MUC5AC in culture supernatants. A similar response was noted in cultures generated from 22-week-old WT mice. However, such increased release of MUC5AC was inhibited when goblet cell cultures were derived from 22-week-old TSP-1 null mice, suggesting a loss of their secretory ability. These results implicate active ocular surface inflammation in older TSP-1 null mice as a potential cause of the disrupted secretory function of goblet cells.

### 3.4. Expression of Cytokine Receptors in Primary Cultures of Conjunctival Goblet Cells

**Figure 4:**
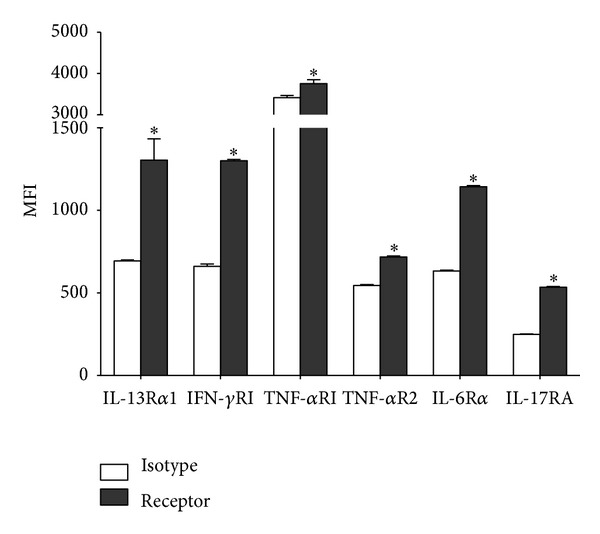
Expression of cytokine receptors in primary cultures of conjunctival goblet cells. The expression of the cytokine receptors IL-13R*α*1, IFN-*γ*RI, TNF-*α*R1, TNF-*α*R2, IL-6R*α*, and IL-17RA was detected using flow cytometry in primary cultures of conjunctival goblet cells. Results are presented as mean fluorescence intensity (MFI) for the cytokine receptors (black bars) and the corresponding isotype controls (white bars). *P < 0.05.

During ocular surface inflammation the conjunctival cytokine environment in TSP-1 null mice includes Th1 (TNF*α*, IFN*γ*) and Th17 (IL-17A, IL-6) besides Th2 cytokines, and these are largely detectable by 6 weeks of age with a progressive increase by 12 weeks [17]. To determine if these cytokines may inhibit the secretory function of TSP-1 null goblet cells as detected in our results, we first assessed the presence of receptors for the cytokines that were detected in TSP-1 null conjunctiva during ocular surface inflammation. Cultured goblet cells from WT mice were stained with immunofluorescence-conjugated antibodies for the indicated receptors and stained cells were analyzed by flow cytometry. Expression of each receptor was evaluated by comparing mean fluorescence intensity (MFI) of the staining with receptor-specific antibody to that of a corresponding isotype control antibody. As shown in Figure 4, receptors for all the cytokines tested were detectable on goblet cells as indicated by the significantly increased receptor specific MFI. These results suggest that cytokines such as IL-13, IFN-*γ*, TNF-*α*, IL-6, and IL-17 that are detectable during ocular surface inflammation in TSP-1 null mice have the potential to influence goblet cells directly via their respective receptors.

### 3.5. Inflammatory Cytokines Alter MUC5AC Secretion by Goblet Cells in Response to Cholinergic Stimulation

**Figure 5:**
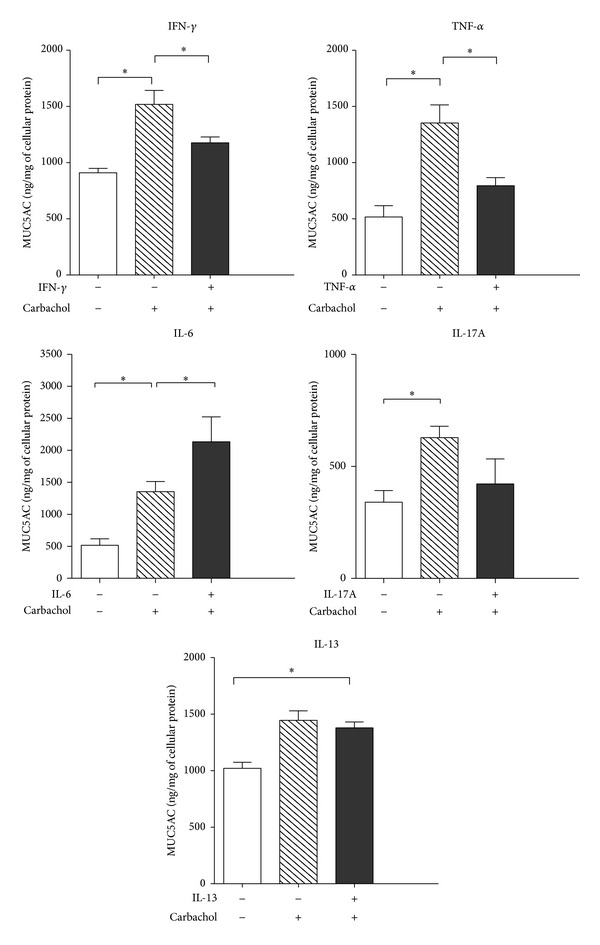
Inflammatory cytokines alter carbachol-mediated MUC5AC secretion by goblet cells. Cultured goblet cells were treated for 24 h with IFN-*γ*, TNF-*α*, IL-6, or IL-17A (10 ng/mL), stimulated with the cholinergic agonist carbachol (10^-3 ^M) for 1 h, and MUC5AC secretion in the supernatants was evaluated by ELISA. *P < 0.05.

To determine if proinflammatory cytokines detected in TSP-1 null conjunctiva may alter the secretory function of goblet cells, we exposed goblet cell cultures derived from WT conjunctiva explants to Th1 (IFN-*γ* and TNF-*α*), Th2 (IL-13), and Th17 (IL-17A and IL-6) cytokines for 24 hr, prior to their cholinergic stimulation with carbachol. As shown in Figure 5, carbachol-mediated mucin secretion of goblet cells exposed to both Th1 cytokines (IFN-*γ* and TNF-*α*) was significantly reduced as compared to those secreted by untreated cells. In contrast carbachol-induced secretion was significantly enhanced if cells were treated with IL-6. Carbachol stimulation of IL-17A or IL-13 exposed goblet cells did not alter their mucin secretion in comparison to untreated goblet cells.

These results suggest that during ocular inflammation, presence of Th1 cytokines in the tissue environment could directly inhibit goblet cell secretory function. These results are consistent with reduced levels of MUC5AC detected in pilocarpine-induced tears (cholinergic stimulation) collected from TSP-1 null mice with ocular surface inflammation as compared to those from WT control mice [17]. Together, our results suggest that in an inflammatory environment in TSP-1 null conjunctiva the inhibitory effects of Th1 cytokines predominate over the enhancing effect of IL-6 on goblet cell secretory function.

### 3.6. Inflammatory Cytokines Alter Goblet Cell Proliferation and Apoptosis

Changes in goblet cell numbers in TSP-1 null mice range from a significant increase during the early stages of ocular inflammation between 8 and 12 weeks to a significant decline with the disease progression by 15 weeks of age [16, 17]. Similar changes are reported in the conjunctiva of SS patients [24, 25]. The basis of these observed changes in goblet cell numbers remains unclear especially since the densities are determined based on the mucin content of goblet cells as detected by Alcian Blue and Periodic Acid Schiff (AB/PAS) staining. It is not clear whether the increase in the number of goblet cells is due to inhibited mucin release or actual proliferation of these cells. Similarly, it remains unclear whether the loss of goblet cells represents a mere inability to detect them by AB/PAS staining after complete release of their mucin content or whether the cell loss is due to cell death.

**Figure 6:**
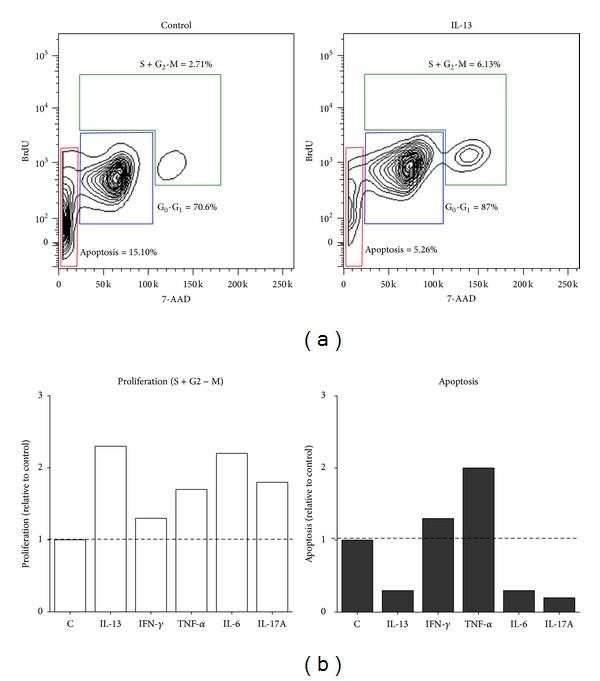
Inflammatory cytokines alter proliferation and apoptosis rate of cultured conjunctival goblet cells. Cultured goblet cells were pulsed with BrdU and treated for 24 h with 10 ng/mL of IL-13, IFN-*γ*, TNF-*α*, IL-6, or IL-17A. Cells stained with fluorescence-conjugated anti-BrdU antibody and viability dye 7-AAD was analyzed by flow cytometry to identify stages of cell cycles. (a) Representative flow cytometry plots are shown and percentage of cells in each of the G_0_-G_1_, S + G_2_-M phases and apoptotic cells are indicated for untreated control and IL-13 treated cells. (b) Bar graphs show changes in proliferation (S + G_2_-M phases) and apoptosis relative to untreated controls.

To address some of these possibilities we evaluated proliferation and apoptotic cell death in goblet cells treated with selected cytokines. We treated goblet cell cultures derived from WT conjunctiva explants with Th1 (IFN-*γ* or TNF-*α*), Th2 (IL-13), and Th17 (IL-17A or IL-6) cytokines and pulsed these cultures with BrdU, as described in methods. Nuclear incorporation of BrdU was detected using fluorescence conjugated anti-BrdU antibody in combination with vital dye 7-AAD. Flow cytometric staining pattern of BrdU and 7-AAD was examined to identify cell cycle stages. Gates were set to identify and enumerate proliferating cells (S + G_2_), resting cells (G_0_ + G_1_), and apoptotic cells. As shown in Figure 6(a), among goblet cells exposed to IL-13 as compared with untreated cultures, a more than twofold increase in proliferating cells (6.13% versus 2.71%) was detected with nearly a threefold reduction in apoptotic cells (5.26% versus 15.1%). These results are consistent with the role of IL-13 described in the differentiation and proliferation of goblet cells in other tissues [10, 26]. Contrary to such an effect of the Th2 cytokine, as shown in Figure 4(b), a relatively lesser degree (<twofold) of proliferation was detected among goblet cells treated with Th1 cytokines (IFN-*γ*: 3.54% and TNF-*α*: 4.56% versus 2.71%), which was however, accompanied with increased apoptotic cells (IFN-*γ*: 30.1% and TNF-*α*: 19.8% versus 15.1%). These results are consistent with the inhibitory effect of Th1 cytokines detected on mucin secretion of goblet cells. The effect of Th17 cytokines resembled that of IL-13 in that an increased proportion of proliferating goblet cells (IL-6: 5.95% and IL-17A: 4.87% versus 2.71%) with reduced apoptotic cells (IL-6: 4.53% and IL-17A: 3.55% versus 15.1%) were detected.

Together our results suggest that inflammatory cytokines indeed alter proliferation as well as apoptosis of conjunctival goblet cells in that increased expression of IL-13 and IL-6 in the conjunctiva may contribute to an initial increase in goblet cell numbers (as seen in TSP-1 null mice) by inducing their proliferation, while goblet cell loss may be attributed to Th1 cytokines that cause their apoptosis. Further investigation is needed to determine whether the inhibitory effect of cytokines on goblet cell mucin secretion leads to their apoptotic cell death.

## 4. Discussion

Ocular surface inflammation in TSP-1 deficient mice is characterized by tissue expression of Th1, Th17, and Th2 cytokines and loss of secretory function with alteration in numbers of mucin secreting goblet cells [17]. In this present study, we developed a primary culture of mouse goblet cells from conjunctival tissue and evaluated the effects of inflammatory cytokines on goblet cells with respect to processes such as mucin secretion, proliferation, and apoptosis. We demonstrated that inflammation has an essential role in the disruption of conjunctival goblet cell functions, and that the proinflammatory cytokines expressed in TSP-1 null conjunctiva induced significant changes in proliferation, apoptosis, and mucin secretion of these cells.

In our experiments Th1 cytokines IFN-*γ* and TNF-*α* inhibited cholinergic stimulus induced mucin secretion and led to goblet cell apoptosis. Significantly increased expression for these cytokines is detectable in TSP-1 deficient conjunctiva as compared to WT tissues, and this expression progressively increases with age similar to that of IL-13 [17]. Such increase was also accompanied with reduced MUC5AC levels in tears secreted in response to cholinergic stimulation in TSP-1 null mice compared to age-matched WT controls. In SS patients, overexpression of both Th1 cytokines is noted in the conjunctiva, which is also correlated with a decline in goblet cell numbers [27, 28]. Similar decline in goblet cells is also detected in TSP-1 null conjunctiva [17]. Thus, our *in vitro* findings are consistent with *in vivo* results and hence support a role for Th1 cytokines in inflammation-mediated conjunctival goblet cell loss during SS. Furthermore, inhibition of mucin secretion by these cytokines also explains reduced tear MUC5AC detected in TSP-1 null mice as well as SS patients [17, 29]. The inhibitory and proapoptotic effect of IFN-*γ* and TNF-*α* in conjunctival goblet cells are similar to that reported in airway goblet cells [30, 31]. However, these reports are based on mucous staining of the cells and do not address secretory aspects of mucins. Studies that examined mucous secretion from intestinal colonic goblet cell lines reported stimulatory effect of IFN-*γ* and TNF-*α* on mucin secretion [32, 33]. These results differ from our observations that address stimulated mucin secretion as against basal levels examined in colonic goblet cells. Furthermore, differential effect of these cytokines on intestinal epithelial apoptosis was reported with its induction by TNF-*α* but not IFN-*γ* [34]. These results clearly indicate tissue specific differences in responses to inflammatory cytokines.

Chronic inflammatory responses in many autoimmune diseases, including SS, involve Th17 cytokines IL-17 and IL-6 [35–38]. Increased mucin expression by airway epithelial cells exposed to these cytokines *in vitro* was correlated with the mucus hypersecretion detected in chronic airway diseases [3]. In our experiments, while both IL-6 and IL-17 induced proliferation of conjunctival goblet cells, only IL-6 enhanced mucin secretion induced by cholinergic stimulus. Considering reduced tear MUC5AC levels in TSP-1 null mice, our *in vitro* results suggest that the stimulatory effect of IL-6 is likely countered by some inhibitory signal *in vivo*. Temporal analysis of TSP-1 deficient conjunctiva has indicated significantly increased expression of TNF-*α* and IFN-*γ* until 12 weeks of age [17]. Therefore, possibly the inhibitory effects of the latter two cytokines predominate over stimulatory effects of IL-6. It is also possible that goblet cell proliferation in response to Th17 cytokines represents a mechanism to replenish lost goblet cells in the conjunctiva. The relative expression of cytokines and tear MUC5AC levels at later stages of the disease may provide further insights into chronic inflammatory processes in Sjögren's pathology.

The expression of the representative Th2 cytokine IL-13 was detected in exocrine gland and peripheral blood mononuclear cells from patients with primary SS [22, 39]. Consistent with these reports, our results also point to an involvement of Th2-mediated pathology in the ocular surface inflammation in TSP-1 null mice, with increased expression of the Th2-associated transcription factor GATA3 and the inflammatory cytokine IL-13 in TSP-1 null conjunctiva. The effects of IL-13 on goblet cell hyperplasia have been extensively studied in the gastrointestinal and respiratory tracts. It is known to induce airway goblet cell differentiation, hyperplasia, and mucus hypersecretion in different inflammatory diseases [10]. In the intestinal epithelium, mucin (MUC5AC) secretion induced by IL-13 is critical for worm expulsion during enteric nematode infections [40, 41]. Although the effect of IL-13 on regulation of conjunctival goblet cell density is not completely understood, the fact that IL-13 deficient mice have a significantly lower number of filled conjunctival goblet cells than wild type mice [42] suggested a potential role of IL-13 in regulating conjunctival goblet cells. In this study, we show that IL-13 has a direct effect on stimulating conjunctival goblet cell proliferation without affecting their mucin secretion. Increased expression of IL-13 in TSP-1 null conjunctiva coincides with an initial increase in goblet cell numbers between 8 and 12 weeks of age prior to their decline [16]. This change is also concurrent with significantly reduced tear MUC5AC levels in TSP-1 null mice [17]. Together our results indicate that although conjunctival goblet cells may resemble airway goblet cells in their hyperplastic response to IL-13, this proliferation may not be accompanied by mucin hypersecretion in tears.

Goblet cell density is a critical parameter that reflects the overall health of the ocular surface [43]. Alterations in mucin secretion and goblet cell number are prominent features of ocular surface diseases [7–9]. The information about the mechanisms leading to goblet cell changes is limited, because it is often extrapolated from studies using whole conjunctival tissue. Goblet cell cultures derived from airway epithelia of hamsters, rats, and humans have been in use for several years in addition to colonic neoplastic cell lines [44–48], but tissue specific differences among goblet cells cannot be overlooked. Unlike conjunctival mucosa, goblet cells in the lungs are not abundant under normal conditions but are induced by a variety of inflammatory stimuli to differentiate them from pulmonary epithelial cell type, Clara cells [49]. In the gastrointestinal mucosa goblet, cell numbers and mucin secretion are modulated by intestinal and colonic microbes [50]. No equivalent alterations in conjunctival goblet, cells have been noted as yet.

Shatos et al. reported the first conjunctival goblet cell culture, achieving the isolation and growth of goblet cells from rat and human conjunctiva [18, 19]. Our study demonstrates that conjunctival goblet cells can be also isolated from mouse conjunctiva using the same explant culture system, retaining goblet cell specific markers and functional activity. This culture serves as an *in vitro* model to study the effect of inflammation on goblet cells in a direct, controlled, and reproducible manner.

## 5. Conclusion

This study demonstrates that inflammatory cytokines associated with the ocular manifestation of Sjögren's syndrome contribute to the pathology by inducing apoptosis and altering mucin secretion and proliferation of conjunctival goblet cells. In addition, this study demonstrates successful and consistent generation of mouse conjunctival goblet cell primary culture for *in vitro* studies.

## References

[1] Davis CW, Dickey BF (2008). Regulated airway goblet cell mucin secretion. *Annual Review of Physiology*.

[2] Ordoñez CL, Khashayar R, Wong HH (2001). Mild and moderate asthma is associated with airway goblet cell hyperplasia and abnormalities in mucin gene expression. *American Journal of Respiratory and Critical Care Medicine*.

[3] Chen Y, Thai P, Zhao YH, Ho YS, DeSouza MM, Wu R (2003). Stimulation of airway mucin gene expression by interleukin (IL)-17 through IL-6 paracrine/autocrine loop. *Journal of Biological Chemistry*.

[4] McCormick DA, Horton LWL, Mee AS (1990). Mucin depletion in inflammatory bowel disease. *Journal of Clinical Pathology*.

[5] Kim YS, Ho SB (2010). Intestinal goblet cells and mucins in health and disease: recent insights and progress. *Current Gastroenterology Reports*.

[6] Gipson IK, Inatomi T, Sulliva DA, Dartt DA, Meneray MA (1998). Cellular origin of mucins of the ocular surface tear film. *Lacrimal Gland, Tear Film, and Dry Eye Syndromes 2*.

[7] Nakamura T, Nishida K, Dota A, Matsuki M, Yamanishi K, Kinoshita S (2001). Elevated expression of transglutaminase 1 and keratinization-related proteins in conjunctiva in severe ocular surface disease. *Investigative Ophthalmology and Visual Science*.

[8] Gilbard JP, Rossi SR (1990). Tear film and ocular surface changes in a rabbit model of neurotrophic keratitis. *Ophthalmology*.

[9] Tseng SCG, Hirst LW, Maumenee AE (1984). Possible mechanisms for the loss of goblet cells in mucin-deficient disorders. *Ophthalmology*.

[10] Kondo M, Tamaoki J, Takeyama K, Nakata J, Nagai A (2002). Interleukin-13 induces goblet cell differentiation in primary cell culture from guinea pig tracheal epithelium. *American Journal of Respiratory Cell and Molecular Biology*.

[11] Ford JG, Rennick D, Donaldson DD (2001). IL-13 and IFN-*γ*: interactions in lung inflammation. *Journal of Immunology*.

[12] Jarry A, Merlin D, Velcich A, Hopfer U, Augenlicht LH, Laboisse CL (1994). Interferon-*γ* modulates cAMP-induced mucin exocytosis without affecting mucin gene expression in a human colonic goblet cell line. *European Journal of Pharmacology: Molecular Pharmacology*.

[13] Saw VPJ, Offiah I, Dart RJ (2009). Conjunctival interleukin-13 expression in mucous membrane pemphigoid and functional effects of interleukin-13 on conjunctival fibroblasts in vitro. *American Journal of Pathology*.

[14] Leonardi A, Fregona IA, Plebani M, Secchi AG, Calder VL (2006). Th1- and Th2-type cytokines in chronic ocular allergy. *Graefe’s Archive for Clinical and Experimental Ophthalmology*.

[15] Yang XO, Seon HC, Park H (2008). Regulation of inflammatory responses by IL-17F. *Journal of Experimental Medicine*.

[16] Turpie B, Yoshimura T, Gulati A, Rios JD, Dartt DA, Masli S (2009). Sjögren’s syndrome-like ocular surface disease in thrombospondin-1 deficient mice. *American Journal of Pathology*.

[17] Contreras-Ruiz L, Regenfuss B, Mir FA, Kearns J, Masli S (2013). Conjunctival inflammation in thrombospondin-1 deficient mouse model of Sjogren's syndrome. *PLoS One*.

[18] Shatos MA, Ríos JD, Horikawa Y (2003). Isolation and characterization of cultured human conjunctival goblet cells. *Investigative Ophthalmology and Visual Science*.

[19] Shatos MA, Rios JD, Tepavcevic V, Kano H, Hodges R, Dartt DA (2001). Isolation, characterization, and propagation of rat conjunctival goblet cells in vitro. *Investigative Ophthalmology and Visual Science*.

[20] Krenzer KL, Freddo TF (1997). Cytokeratin expression in normal human bulbar conjunctiva obtained by impression cytology. *Investigative Ophthalmology and Visual Science*.

[21] Inatomi T, Spurr-Michaud S, Tisdale AS, Zhan Q, Feldman ST, Gipson IK (1996). Expression of secretory mucin genes by human conjunctival epithelia. *Investigative Ophthalmology and Visual Science*.

[22] Mitsias DI, Tzioufas AG, Veiopoulou C (2002). The Th1/Th2 cytokine balance changes with the progress of the immunopathological lesion of Sjogren’s syndrome. *Clinical and Experimental Immunology*.

[23] Jabs DA, Prendergast RA, Campbell AL (2007). Autoimmune Th2-mediated dacryoadenitis in MRL/MpJ mice becomes Th1-mediated in IL-4 deficient MRL/MpJ mice. *Investigative Ophthalmology and Visual Science*.

[24] Ralph RA (1975). Conjunctival goblet cell density in normal subjects and in dry eye syndromes. *Investigative Ophthalmology*.

[25] Nelson JD, Wright JC (1984). Conjunctival goblet cell densities in ocular surface disease. *Archives of Ophthalmology*.

[26] Kanoh S, Tanabe T, Rubin BK (2011). IL-13-induced MUC5AC production and goblet cell differentiation is steroid resistant in human airway cells. *Clinical and Experimental Allergy*.

[27] Pisella PJ, Brignole F, Debbasch C (2000). Flow cytometric analysis of conjunctival epithelium in ocular rosacea and keratoconjunctivitis sicca. *Ophthalmology*.

[28] Brignole F, Pisella PJ, Goldschild M, De Saint Jean M, Goguel A, Baudouin C (2000). Flow cytometric analysis of inflammatory markers in conjunctival epithelial cells of patients with dry eyes. *Investigative Ophthalmology and Visual Science*.

[29] Argüeso P, Balaram M, Spurr-Michaud S, Keutmann HT, Dana MR, Gipson IK (2002). Decreased levels of the goblet cell mucin MUC5AC in tears of patients with Sjögren syndrome. *Investigative Ophthalmology and Visual Science*.

[30] Cohn L, Homer RJ, Niu N, Bottomly K (1999). T helper 1 cells and interferon *γ* regulate allergic airway inflammation and mucus production. *Journal of Experimental Medicine*.

[31] Shi ZOQ, Fischer MJ, De Sanctis GT, Schuyler MR, Tesfaigzi Y (2002). IFN-*γ*, but not fas, mediates reduction of allergen-induced mucous cell metaplasia by inducing apoptosis. *Journal of Immunology*.

[32] Smirnova MG, Birchall JP, Pearson JP (2000). TNF-*α* in the regulation of MUC5AC secretion: some aspects of cytokine-induced mucin hypersecretion on the in vitro model. *Cytokine*.

[33] Jarry A, Muzeau F, Laboisse C (1992). Cytokine effects in a human colonic goblet cell line. Cellular damage and its partial prevention by 5 aminosalicylic acid. *Digestive Diseases and Sciences*.

[34] Juuti-Uusitalo K, Klunder LJ, Sjollema KA (2011). Differential effects of TNF (TNFSF2) and IFN-*γ* on intestinal epithelial cell morphogenesis and barrier function in three-dimensional culture. *PLoS One*.

[35] Afzali B, Lombardi G, Lechler RI, Lord GM (2007). The role of T helper 17 (Th17) and regulatory T cells (Treg) in human organ transplantation and autoimmune disease. *Clinical and Experimental Immunology*.

[36] Nguyen CQ, Hu MH, Li Y, Stewart C, Peck AB (2008). Salivary gland tissue expression of interleukin-23 and interleukin-17 in Sjögren’s syndrome: findings in humans and mice. *Arthritis and Rheumatism*.

[37] Doyle ME, Boggs L, Attia R (2007). Autoimmune dacryoadenitis of NOD/LtJ mice and its subsequent effects on tear protein composition. *American Journal of Pathology*.

[38] Sakai A, Sugawara Y, Kuroishi T, Sasano T, Sugawara S (2008). Identification of IL-18 and Th17 cells in salivary glands of patients with Sjögren’s syndrome, and amplification of IL-17-mediated secretion of inflammatory cytokines from salivary gland cells by IL-18. *Journal of Immunology*.

[39] Villarreal GM, Alcocer-Varela J, Llorente L (1996). Differential interleukin (IL)-10 and IL-13 gene expression in vivo in salivary glands and peripheral blood mononuclear cells from patients with primary Sjögren’s syndrome. *Immunology Letters*.

[40] Hasnain SZ, Evans CM, Roy M (2011). Muc5ac: a critical component mediating the rejection of enteric nematodes. *Journal of Experimental Medicine*.

[41] Khan I, Collins SM (2004). Immune-mediated alteration in gut physiology and its role in host defence in nematode infection. *Parasite Immunology*.

[42] De Paiva CS, Raince JK, McClellan AJ (2011). Homeostatic control of conjunctival mucosal goblet cells by NKT-derived IL-13. *Mucosal Immunology*.

[43] Kinoshita S, Kiorpes TC, Friend J, Thoft RA (1983). Goblet cell density in ocular surface disease. A better indicator than tear mucin. *Archives of Ophthalmology*.

[44] Wu R, Martin WR, Robinson CB (1990). Expression of mucin synthesis and secretion in human tracheobronchial epithelial cells grown in culture. *American Journal of Respiratory Cell and Molecular Biology*.

[45] Wu R, Nolan E, Turner C (1985). Expression of tracheal differentiated functions in serum-free hormone-supplemented medium. *Journal of Cellular Physiology*.

[46] Kaartinen L, Nettesheim P, Adler KB, Randell SH (1993). Rat tracheal epithelial cell differentiation in vitro. *In Vitro Cellular and Developmental Biology—Animal*.

[47] Fogh J, Fogh JM, Orfeo T (1977). One hundred and twenty seven cultured human tumor cell lines producing tumors in nude mice. *Journal of the National Cancer Institute*.

[48] McCool DJ, Forstner JF, Forstner GG (1994). Synthesis and secretion of mucin by the human colonic tumour cell line LS180. *Biochemical Journal*.

[49] Chen G, Korfhagen TR, Xu Y (2009). SPDEF is required for mouse pulmonary goblet cell differentiation and regulates a network of genes associated with mucus production. *Journal of Clinical Investigation*.

[50] Deplancke B, Gaskins HR (2001). Microbial modulation of innate defense: goblet cells and the intestinal mucus layer. *American Journal of Clinical Nutrition*.

